# Whole-exome sequencing to identify somatic mutations in peritoneal metastatic gastric adenocarcinoma: A preliminary study

**DOI:** 10.18632/oncotarget.9707

**Published:** 2016-05-30

**Authors:** Hao Liu, Fengping Li, Yu Zhu, Tingting Li, Haipeng Huang, Tian Lin, Yanfeng Hu, Xiaolong Qi, Jiang Yu, Guoxin Li

**Affiliations:** ^1^ Department of General Surgery, Nanfang Hospital, Southern Medical University, Guangzhou, Guangdong, China

**Keywords:** gastric cancer, peritoneal metastasis, whole-exome sequencing (WES), Sanger sequencing, somatic mutation

## Abstract

Peritoneal metastasis occurs in more than half of patients with unresectable or recurrent gastric cancer and is associated with the worst prognosis. The associated genomic events and pathogenesis remain ambiguous. The aim of the present study was to characterize the mutation spectrum of gastric cancer with peritoneal metastasis and provide a basis for the identification of new biomarkers and treatment targets. Matched pairs of normal gastric mucosa and peritoneal tissue and matched pairs of primary tumor and peritoneal metastasis were collected from one patient for whole-exome sequencing (WES); Sanger sequencing was employed to confirm the somatic mutations. G>A and C>T mutations were the two most frequent transversions among the somatic mutations. We confirmed 48somatic mutations in the primary site and 49 in the peritoneal site. Additionally, 25 non-synonymous somatic variations (single-nucleotide variants, SNVs) and 2 somatic insertions/deletions (INDELs) were confirmed in the primary tumor, and 30 SNVs and 5 INDELs were verified in the peritoneal metastasis. Approximately 59% of the somatic mutations were shared between the primary and metastatic site. Five genes (TP53, BAI1, THSD1, ARID2, and KIAA2022) verified in our study were also mutated at a frequency greater than 5%in the COSMIC database. We also identified 9genes (ERBB4, ZNF721, NT5E, PDE10A, CA1, NUMB, NBN, ZFYVE16, and NCAM1) that were only mutated in metastasis and are expected to become treatment targets. In conclusion, we observed that the majority of the somatic mutations in the primary site persisted in metastasis, whereas several single-nucleotide polymorphisms occurred de novo at the second site.

## INTRODUCTION

Gastric cancer is the fifth most commonly diagnosed cancer and the third leading cause of cancer mortality worldwide [1–3]. Although a steady decline in cancer incidence and mortality have been observed in recent years, an estimated 951,600 new gastric cancer cases and 723,100 deaths were reported in 2012. Approximately 40% of gastric cancer cases occur in China, and many are diagnosed at an advanced stage with a tendency toward metastasis and recurrence [4]. Peritoneal carcinoma occurs in both the advanced and early stages of gastric cancer and is the most common type of metastasis and recurrence [5–7]. The median survival of patients with peritoneal metastasis is less than 6 months due to the development of resistance to therapy [8–11]. Peritoneal metastasis accounts for 20.0–53.5% of recurrences after radical resection for gastric cancer [12]. Elucidating the molecular mechanism driving peritoneal metastasis of gastric adenocarcinoma is thus critical.

Next-generation sequencing has emerged as a powerful tool to identify potential oncogene targets for personal therapeutic intervention as part of precision medicine and has revolutionized cancer research [13, 14]. Whole-genome sequencing provides a relatively unbiased review of the genome but is costly and produces large datasets, which present a heavy computational burden. Thus, many researchers and clinicians choose whole-exome sequencing (WES) for personal management [15]. WES directly sequences all exonic regions, which account for only 1% of the whole genome, to accurately depict the relationship between mutations and phenotypes. Furthermore, WES can achieve higher sequencing depth at a lower cost than whole-genome sequencing [16, 17]. In the era of precision medicine, WES is approved to facilitate the identification of candidate predictive biomarkers of response in metastatic cancer harboring biologically informative alterations [18].

Gastric adenocarcinoma is the most common type of gastric cancer. Once peritoneal metastasis is observed, surgery is no longer preferred as the therapeutic strategy, leading to difficulty in collecting matched primary and metastatic tumor specimens and a lack of relevant reports. Xia and colleagues recently suggested that non-curative dissection of peritoneal metastasis in selected gastric cancer patients significantly prolongs survival [19, 20]. Accordingly, we collected matched specimens for WES after non-curative dissection [21]. The genomic events during gastric cancer dissemination to the peritoneum are unknown. In our study, we aimed to reveal the mutation spectrum of peritoneal metastatic gastric adenocarcinoma by WES. Normal gastric mucosa, primary cancer, normal peritoneum and peritoneal metastasis tissues were collected from one patient.

## RESULTS

### Identification of single-nucleotide variants (SNVs) and insertions/deletions (INDELs) in primary and secondary tumor sites

**Figure 1:**
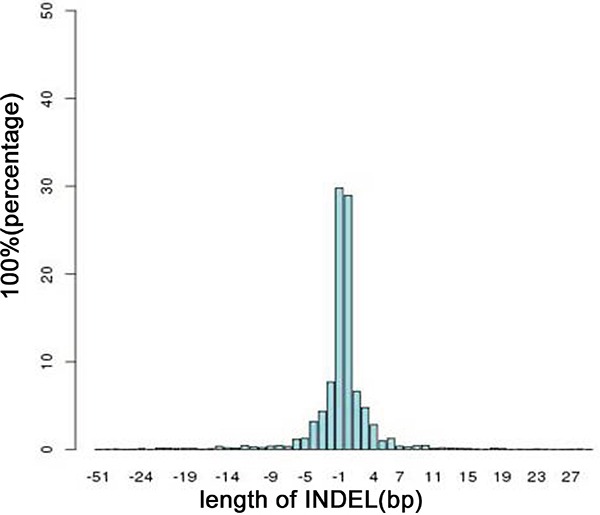
Distribution of the lengths of all INDELs in the on-target regions A length >0 indicates an insertion mutation; otherwise, the length represents a deletion mutation.

We identified 46,609 SNVs and 48,215 SNVs in the primary gastric cancer and peritoneal metastasis, respectively; approximately 89% of the SNVs had been detected by the 1000 Genomes Project. Additionally, 4,506 INDELs and 4,643 INDELs were identified in the primary gastric cancer and peritoneal metastasis, respectively, of which 43% had been identified by the 1000 Genomes Project. Even the normal gastric mucosa and peritoneum had many SNVs and INDELs. Most of the INDELs were less than five bases in length; small INDELs represent previous in-frame shift mutations (Figure 1).

### Identification of somatic variations from primary gastric cancer and peritoneal metastasis

**Figure 2:**
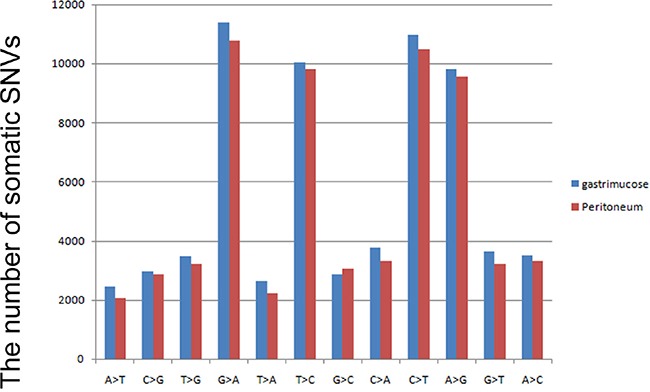
Mutation spectrum of non-synonymous variations in gastric cancer The blue bar represents somatic mutations in primary gastric cancer, and the red bar indicates somatic non-synonymous mutations identified in peritoneal metastasis.

By comparing the normal gastric mucosa and peritoneal tissue, we identified 48 non-synonymous somatic mutations in the primary tumor and 49 non-synonymous somatic mutations in the peritoneal metastasis after filtering, including7 somatic INDELs in the primary gastric cancer and 11 somatic INDELs in the secondary carcinoma. In the primary gastric adenocarcinoma, most (gastric adenocarcinoma vs. peritoneal nodules: 39%vs.40%, respectively) of the on-target somatic mutations were in the exonic region, and most (gastric adenocarcinoma vs. peritoneal nodules: 20% vs. 20%%, respectively) were synonymous somatic mutations. The primary gastric cancer and peritoneal metastasis exhibited similar mutation spectra. We observed that G: C>A: T was the most frequent transversion in somatic mutations (Figure 2). The transversion of C: G>T: A was also enriched in the somatic mutations, consistent with a report by Chen et al. [14].

### Confirmation of somatic non-synonymous mutations

**Figure 3:**
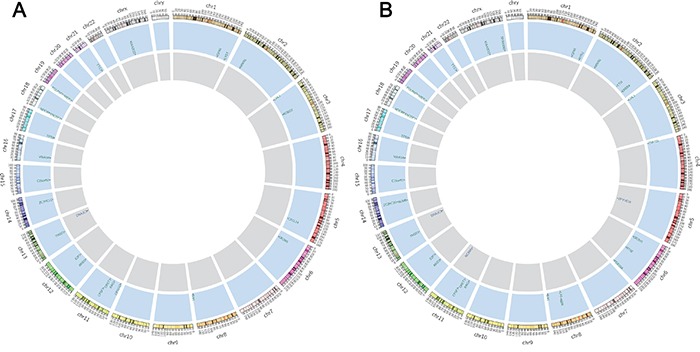
Non-synonymous somatic mutations discovered by WES From outside to inside: the outer ring represents the chromosome number and section, the blue ring and letters indicate mutated genes with non-synonymous somatic variations and their chromosomal locations, and the gray ring shows genes with somatic INDEL mutations. Figure 3A and 3B summarize the confirmed non-synonymous somatic mutations in primary gastric adenocarcinoma and peritoneal metastasis, respectively.

**Figure 4:**
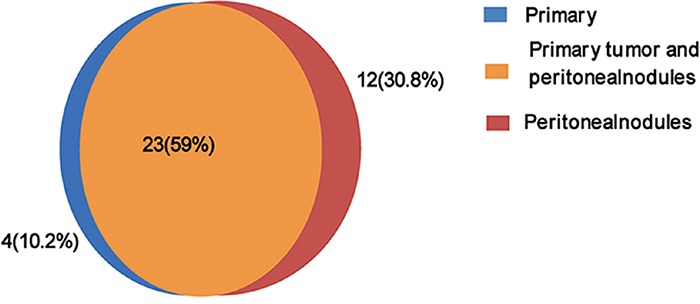
Distribution of confirmed non-synonymous somatic mutations in primary gastric cancer and peritoneal metastasis

Twenty-five non-synonymous somatic SNVs and 2 somatic INDELs were confirmed in the primary tumor (Table 1), and thirty non-synonymous SNVs and five INDELs were verified in the peritoneal metastasis (Table 2) (Figure 3). Approximately 59% of the somatic mutations were shared between the primary and metastatic sites (Figure 4). The 22 mutated genes in common were IGFN1, NRXN1, CHL1, OR2W1, BAI1, RAG1, OR5T1, CPSF7, ARID2, THSD1, VWA3A, ZC2HC1C, C15orf57, TP53, ENOSF1, NDC80, MIER2, POLRMT, TTC3, KIAA2022, DNAJC3, and E2F7. Wealso identified nine genes (ERBB4, ZNF721, NT5E, PDE10A, CA1, NUMB, NBN, ZFYVE16, and NCAM1) that were only mutated in metastasis. Searching the KEGG PATHWAY database revealed that two genes (TP53 and BAI) that are important members of theTP53 pathway were simultaneously mutated in the gastric cancer and peritoneal metastasis. Both tumor sites also harbored two somatic non-synonymous mutations in the TP53 gene.

**Table 1:** Somatic mutations identified by WES in primary gastric cancer

Chr	Position	Exon	Allele change	Amino acid variation	Certification	Gene	db SNP	Polyphen2 prediction
chr14	75537380	exon2	A>G	Y35C	Y	ZC2HC1C	−	Benign
chr15	40846283	exon4	C>A	A158S	Y	C15orf57	−	Damaging
chr1	111957501	exon11	24bp del	533_541del	N	OVGP1	rs201350653	−
chr2	179582678	exon83	A>G	V7108A	N	TTN	−	Benign
chr17	7574026	exon6	C>A	G202V	Y	TP53	−	Damaging
chr17	7574027	exon6	C>A	G202W	Y	TP53	−	Damaging
chr19	9057778	exon3	A>C	S9890A	N	MUC16	−	Probably damaging
chr1	103481225	exon10	G>A	P380L	N	COL11A1	−	Damaging
chr2	212248608	exon27	T>C	E1204G	N	ERBB4	−	Probably damaging
chr17	10427078	exon36	C>G	D1767H	N	MYH2	−	Damaging
chr3	77684050	exon24	C>G	R1264G	Y	ROBO2	−	Benign
chrX	73961529	exon3	C>G	D955H	Y	KIAA2022	−	Damaging
chr1	235926118	exon22	A>T	I2052N	Y	LYST	−	Probably damaging
chr12	46242737	exon13	T>C	F567L	Y	ARID2	−	Damaging
chr3	30691871	exon3	1bp ins	E125fs	N	TGFBR2	−	−
chr13	52972327	exon3	G>T	L21I	Y	THSD1	−	Probably damaging
chr8	143545787	exon1	C>A	D76E	Y	BAI1	−	Damaging
chr3	439979	exon24	C>G	P1039R	Y	CHL1	−	Damaging
chr1	38185137	exon15	T>A	K902M	N	EPHA10		Damaging
chr2	50280649	exon4	C>A	Q231H	Y	NRXN1	−	Damaging
chr11	36595492	exon2	T>C	I213T	Y	RAG1	−	Benign
chr12	77449826	exon3	1 bp ins	F60fs	Y	E2F7	−	−
chr11	36595553	exon2	1 bp ins	S233fs	N	RAG1	−	−
chr21	38494232	exon12	T>C	L339P	Y	TTC3	−	Damaging
chr4	6304013	exon8	G>G	G831C	N	WFS1	−	Damaging
chr11	14880776	exon13	C>A	A903E	N	PDE3B	−	Damaging
chr15	23931941	exon1	G>C	R142G	N	NDN	−	Probably damaging
chr6	165801813	exon17	A>C	F596V	N	PDE10A	−	Damaging
chr1	201168763	exon7	C>T	R147W	Y	IGFN1	−	−
chr5	178392069	exon5	G>T	G222W	N	ZNF454	−	Damaging
chr22	29885858	exon4	18bp del	744_749del	N	NEFH	rs59890097	−
chr8	90958479	exon13	1 bp ins	K653fs	N	NBN		−
chr11	56043937	exon1	A>C	S275R	Y	OR5T1	−	Benign
chr19	620049	exon12	G>A	A932V	Y	POLRMT	−	Damaging
chr4	437571	exon3	C>G	A229P	N	ZNF721	−	Probably damaging
chr18	697265	exon3	A>C	L95R	Y	ENOSF1	−	Damaging
chr11	4936758	exon1	C>A	G46C	Y	OR51G2	−	Damaging
chr18	2577827	exon4	G>A	D88N	Y	NDC80	−	Damaging
chr19	308907	exon11	C>T	G335S	Y	MIER2	−	Damaging
chrX	142596888	exon2	T>G	K61T	N	SPANXN3	−	Benign
chr13	96439336	exon11	3 bp del	429_429del	Y	DNAJC3	−	−
chr16	22111606	exon4	T>C	L106S	Y	VWA3A	−	Damaging
chr6	29012638	exon1	C>T	M105I	Y	OR2W1	−	Probably damaging
chr16	88600174	exon10	G>A	R603H	N	ZFPM1	−	Damaging
chr14	73822447	exon4	G>A	R5W	N	NUMB	−	Damaging
chr8	86241943	exon6	T>A	E215V	N	CA1	−	Probably damaging
chr5	134910301	exon3	C>T	R94H	Y	CXCL14	rs139612389	Damaging
chr11	61179326	exon8	G>A	R390C	Y	CPSF7		Damaging

**Table 2:** somatic mutations identified by WES in secondary cancer site

Chr	Position	Exon	Allele change	Amino acid variation	Certification	Gene	db SNP	Polyphen2 prediction
chr1	38185137	exon15	T>A	K902M	N	EPHA10		Damaging
chr1	103481225	exon10	G>A	P380L	N	COL11A1		Damaging
chr1	201168763	exon7	C>T	R147W	Y	IGFN1		−
chr2	237619910	exon16	A>T	N496I	Y	RYR2		Benign
chr2	50280649	exon4	C>A	Q231H	Y	NRXN1		Benign
chr2	179582678	exon83	A>G	V7108A	Y	TTN		Benign
chr3	212248608	exon27	T>C	E1204G	Y	ERBB4		Probably damaging
chr3	439979	exon24	C>G	P1039R	Y	CHL1		Damaging
chr4	75786223	exon5	A>T	F851I	N	ZNF717		Damaging
chr4	437571	exon3	C>G	A229P	Y	ZNF721		Probably damaging
chr6	6304013	exon8	G>T	G831C	N	WFS1		Damaging
chr6	29012638	exon1	C>T	M105I	Y	OR2W1		Probably damaging
chr6	86199234	exon6	C>T	T376M	Y	NT5E		Probably damaging
chr8	165801813	exon17	A>C	F596V	Y	PDE10A		Damaging
chr8	86241943	exon6	T>A	E215V	Y	CA1		Probably damaging
chr11	143545787	exon1	C>A	D76E	Y	BAI1		Damaging
chr11	4936758	exon1	C>A	G46C	N	OR51G2		Damaging
chr11	36595492	exon2	T>C	I213T	Y	RAG1		Benign
chr11	56043937	exon1	A>C	S275R	Y	OR5T1		Benign
chr12	61179326	exon8	G>A	R390C	Y	CPSF7		Damaging
chr13	46242737	exon13	T>C	F567L	Y	ARID2		Damaging
chr14	52972327	exon3	G>T	THSD1	Y	THSD1		Probably damaging
chr14	73822447	exon4	G>A	R5W	Y	NUMB		Damaging
chr15	75537380	exon2	A>G	Y35C	Y	ZC2HC1C		Benign
chr16	40846283	exon4	C>A	A158S	Y	C15orf57		Damaging
chr16	22111606	exon4	T>C	L106S	Y	VWA3A		Damaging
chr17	88600174	exon10	G>A	R603H	N	ZFPM1		Damaging
chr17	7574026	exon6	C>A	G202V	Y	TP53		Damaging
chr17	7574027	exon6	C>A	G202W	Y	TP53		Damaging
chr18	697265	exon3	A>C	L95R	Y	ENOSF1		Damaging
chr19	2577827	exon4	G>A	D88N	Y	NDC80		Damaging
chr19	308907	exon11	C>T	G335S	Y	MIER2		Damaging
chr19	620049	exon12	G>A	A932V	Y	POLRMT		Damaging
chr21	9057778	exon3	A>C	S9890A	N	MUC16		Probably damaging
chr22	38494232	exon12	T>C	L339P	Y	TTC3		Damaging
chrX	19119441	exon1	G>A	G177R	N	TSSK2		Damaging
chrX	73961529	exon7	C>G	D955H	Y	KIAA2022		Damaging
chr3	142596888	exon9	T>G	K61T	Y	SPANXN3		Benign
chr3	30691871	exon3	1 bp ins	E125fs	N	TGFBR2		−
chr12	77449826	exon3	1 bp ins	F60fs	Y	E2F7		−
chr13	96439336	exon11	3 bp del	429_429del	Y	DNAJC3		−
chr11	36595553	exon2	1 bp ins	S233fs	N	RAG1		−
chr8	90958479	exon13	1bp ins	K653fs	Y	NBN		−
chr6	160211645	exon1	3bp del	9_10del	N	MRPL18	rs58504486	−
chr5	79746372	exon9	4 bp del	1117_1118del	Y	ZFYVE16		−
chr16	70954703	exon46	15bp del	2521_2525del	N	HYDIN	rs67115747	−
chr9	139992321	exon5	1 bp del	L221fs	N	MAN1B1		−
chr11	112832362	exon2	1 bp del	T10fs	Y	NCAM1		−
chr3	75787042	exon5	4 bp ins	L578fs	N	ZNF717		−

### Comparison with a public database

**Figure 5:**
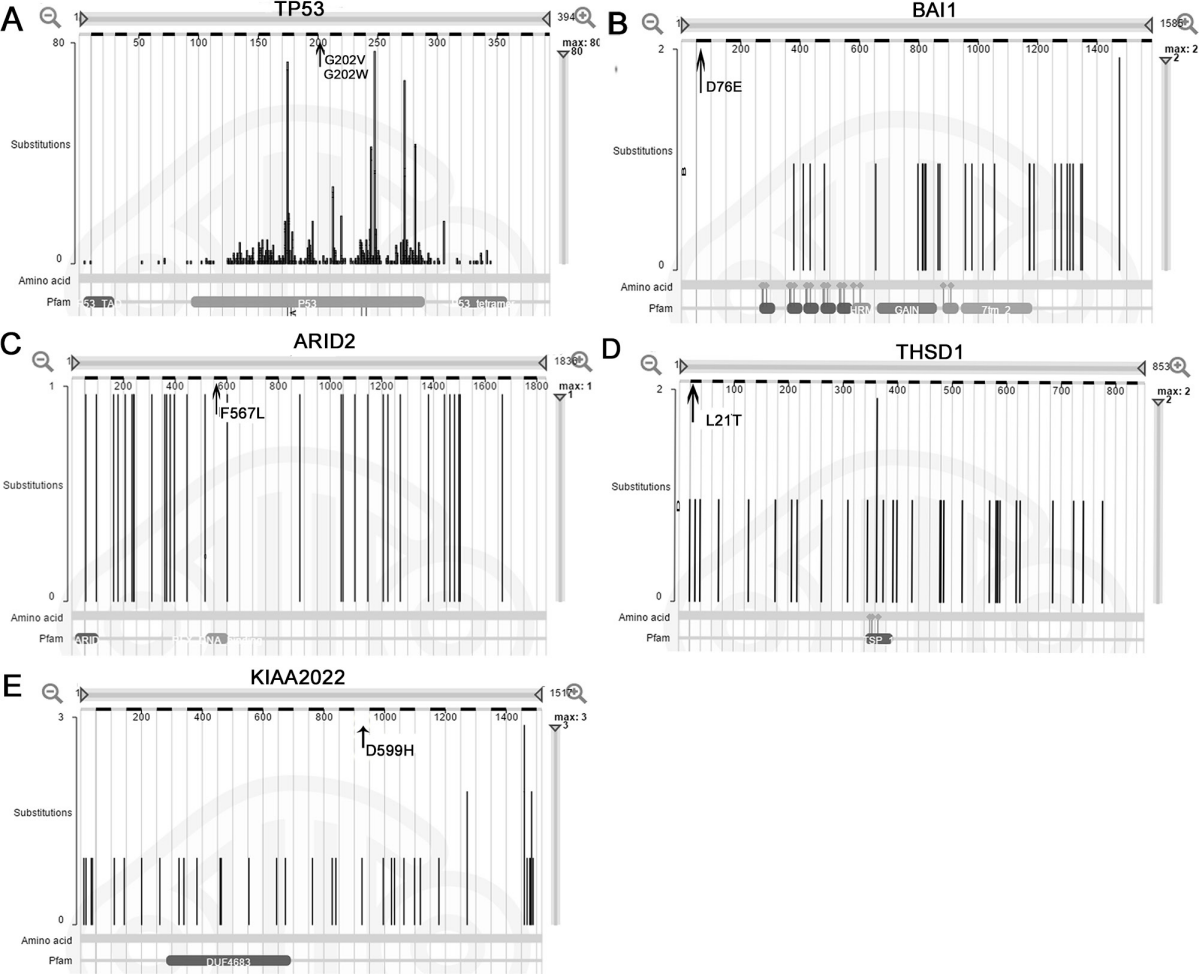
Mutation distribution of the five genes in the COSMIC database A, B, C, D, and E represent the genesTP53, BAI1, ARID2, THSD1and KIAA2022, respectively. The arrow indicates the site of the mutation identified in gastric adenocarcinoma and matched peritoneal metastasis.

In the COSMIC database, the most common type of substitutional somatic mutation in gastric adenocarcinoma is the transversion of G: C> A: T, and the major INDELs are short(less than 5 bases), consistent with our observations of the gastric adenocarcinoma with peritoneal metastasis in the present study. Five genes (TP53, BAI1, THSD1, ARID2, and KIAA2022) verified in our study were also mutated at a frequency greater than 5%in gastric adenocarcinoma in the COSMIC database, but the amino acid changes F567L in ARID2, D76E in BAI1, L21I in THSD1, and D955H in KIAA2022 had not been reported previously in gastric cancer. In the COSMIC database, TP53 was mutated in 33.4% of gastric adenocarcinoma, and 2 point mutations in TP53 were identified in the present study. The ARID2 gene was mutated at a frequency of 5.3% in 514 gastric adenocarcinoma cases. In our study, the patient also had an ARID2 gene missense mutation in both the primary tumor and peritoneal metastasis. The mutation of BAI1 leads to the amino acid mutation L21T and exerts a damaging effect on its function. BAI1 was mutated in approximately 5% of gastric adenocarcinoma in the COSMIC database, and this angiogenesis inhibitor gene was even mutated as highly as 11% among the 295 samples reported in The Cancer Genome Atlas Research Network. THSD1, which is mutated in both the primary gastric cancer and peritoneal metastasis, has a mutation frequency of 5.4%(21/389) in gastric adenocarcinoma in the COSMIC database. KIAA2022 was mutated at a high frequency of 6.9% (27/389) as analyzed by the COSMIC database. In our study, we identified a missense somatic mutation in KIAA2022, and the consequent amino acid variationD955H would presumably exert a damaging effect in the protein (Figure 5).

## DISCUSSION

We performed WES to uncover the somatic mutation landscape of gastric adenocarcinoma with peritoneal metastasis. The mechanism of the peritoneal dissemination of gastric adenocarcinoma remains unknown, and there is no standard therapeutic method, resulting in poor survival [22]. Targeted gene analysis has identified TNF-alpha and EpCAM expression as facilitating peritoneal metastasis, whereas IL-1B might not correlate with the process of metastasis [23, 24]. Catumaxomab, an anti-EpCAM monoclonal antibody, coupled with intraperitoneally administered paclitaxel are recommended to effectively relieve gastric cancer-derived peritoneal metastasis [25]. Zhang et al. recently identified 27 somatic mutated genes and infusion of GPX4 and MPND in the19q13.3-13.4 region by whole-genome and -transcriptome sequencing of one patient [4]. The different mutation events in the present study may be attributable to the different pathological characteristics of the selected specimen. However, we cannot neglect the errors resulting from the small number of specimens in these two studies. Lim et al revealed significant enrichment of mutations in the Rho-ROCK signaling pathway by WES of gastric cancer and matched malignant ascites [21]. The discrepancies between Lim's findings and our results may be due in part to the diversity in sample selection. Genomic alterations also accumulate during the process of metastasis. The heterogeneity of gastric cancer, as revealed by Bass AJ e al., may also underlie the differences between our results and those of other studies [26]. We confirmed 20 somatic SNVs and 2 INDELs in one gastric adenocarcinoma by WES. After searching the KEGG public database, we identified two genes in the p53 pathway. The spontaneous mutation of p53 and BAI1 might play a vital role in the development of peritoneal metastasis of gastric adenocarcinoma. Five genes (TP53, BAI1, ARID2, THSD1, and KIAA2022) mutated in primary cancer and nine genes (ERBB4, ZNF721, NT5E, PDE10A, CA1, NUMB, NBN, ZFYVE16, NCAM1) mutated in peritoneal metastasis may be targets for the effective treatment of peritoneal metastatic gastric adenocarcinoma.

Somatic mutations of BAI1 and its family members BAI2 and BAI3, which encode brain-specific angiogenesis inhibitors, have been identified in several cancers, including breast cancer, lung cancer, and ovarian cancer. Only BAI1 is transcriptionally regulated by p53 [27]. BAI1 is reported to be expressed in gastric epithelia during *Helicobacter pylori* infection and mediates the engulfment of apoptotic gastric epithelial cells [28]. BAI1 is also decreased in gastric cancer in patients with distant metastasis and poor prognosis [29]. Therefore, the role of BAI1 in metastatic gastric adenocarcinoma merits further study.

ARID2 is a subunit of the PBAF chromatin-remodeling complex and has been reported to be mutated in melanoma (7%) and colorectal cancer (13%). ARID2 has been suggested to be tumor suppressive in hepatocellular carcinoma, with a mutation rate of 6.5%. ARID2 is mutated in 13% of colorectal cancer patients with microsatellite instability [30]. ARID2is also mutated in 18.2% of hepatocellular carcinoma cases, leading to inactivation of the coding protein [31]. Furthermore, the expression of ARID2 and its family members is lost during gastric cancer progression, but the effect of ARID2 on tumor progression is weaker than that of ARID1A [32]. In our study, ARID2 was synonymously mutated in exon13 and putatively disrupted the protein's function.

THSD1 is located in the chromosome 13q region that is frequently lost in esophageal cancer, accelerating cancer formation [33]. In the COSMIC database, THSD1 was mutated in 5.4% (21/389) of gastric adenocarcinoma. In colorectal cancer, THSD1 is down-regulated and methylated in the promoter region [34], but the expression of THSD1 in gastric cancer with peritoneal metastasis and its role in turmorigenesis are not yet known. The THSD1 mutation in exon3 observed in this study results in the amino acid change L21T, likely exerts a damaging effect on the protein's function.

KIAA2022 is a G-protein-coupled purinergic receptor gene located in the pseudoautosomal region of the X chromosome. For gastric adenocarcinoma, theKIAA2022 gene is mutated at a high frequency of 6.9% (27/389) in the COSMIC database. ZC2HC1C and C15orf57 were mutated in both the primary gastric cancer tissue and peritoneal metastasis and identified in gastric cancer for the first time. The mutation inZC2HC1C was an A>G substitution in exon 2, leading to a putatively benign effect, whereas the C15orf57 somatic mutation likely exerted a damaging effect on the protein.

Nine genes were mutated in the peritoneal metastasis: ERBB4, ZNF721, NT5E, PDE10A, CA1, NUMB, NBN, ZFYVE16, and NCAMI. The acquisition of somatic mutations in these genes after metastasis, may drive phenotypic changes in cancer cells and the metastasis of gastric adenocarcinoma. As a member of the EGFR family, ERBB4 is frequently activated in brain metastases and metastatic colorectal cancer [35, 36]. In breast cancer, ERBB4plays an important role in the survival of ERBB2+ cells after they acquire resistance to lapatinib and trastuzumab [37]. The druggable gene ERBB4 was also reported to be mutated in malignant ascites of patients with gastric cancer [21]. We identified a somatic mutation in ERBB4 that may exert a damaging effect. Whether ERBB4 is indispensable for facilitating the peritoneal metastasis of gastric adenocarcinoma requires further investigation. The NUMB protein participates in the control of asymmetric division, ubiquitination of transcriptional factor p53, and endocytosis of the Notch receptor, and NUMB mutation leads to several types of cancer [38, 39]. We will further investigate the role of NUMB in peritoneal metastatic gastric cancer in a future study. The effects of somatic mutations in genes encoding zinc finger proteins (ZNF721 and ZFYVE16), a purine and pyrimidine metabolism protein (NT5E), carbonic anhydrase(CA1), and cyclic nucleotide phosphodiesterase(PDE10A)on the progress of gastric cancer requires further study. NCAM1, also known as cell adhesion molecular CD56, plays an important role in immune surveillance for the expansion of T cells, andNCAM1 over-expression in Ewing sarcoma indicates poor prognosis [40]. An insertion mutation was validated in the DNA repair gene NBN, which is altered in high-risk breast cancer [41]. Zhou et al. reported that polymorphic NBN tended to improve chemotherapeutic outcomes in gastric cancer. Because DNA repair capacity is attenuated during the evolution of cancer, more phenotypic changes tend to accelerate the formation of metastasis.

In summary, we performed WES of a gastric adenocarcinoma with peritoneal metastasis. Five genes (TP53, BAI1, THSD1, ARID2, and KIAA2022) identified as frequently mutated in the COSMIC database may drive gastric adenocarcinoma dissemination to the peritoneum. The effect of the two novel somatic mutated genes (ZC2HC1C and C15orf57) in gastric cancer requires further investigation. Nine genes (ERBB4, ZNF721, NT5E, PDE10A, CA1, NUMB, NBN, ZFYVE16, and NCAMI) mutated in peritoneal metastasis are potential molecular targets for the treatment of metastatic gastric cancer. The major limitations of our study are the small sample size and single sequencing platform.

## MATERIALS AND METHODS

### Clinicopathology of the patient

The 60-year-old male patient was diagnosed with gastric cancer with peritoneal metastasis by computed tomography (CT) and gastroscope inspection at Nanfang Hospital of Southern Medical University in 2014. The patient had not received preoperative chemotherapy or radiotherapy; he underwent palliative total gastrectomy and D2-NO.10 lymphadenectomy followed by the construction of Roux-en-Y esophagojejunostomy. After surgery, the specimens were subjected to further pathological testing, which revealed poorly differentiated primary gastric adenocarcinoma located in the cardia of the stomach of Borrmann III type with a maximum diameter of 5 cm. Furthermore, the gastric tumor was of the mixed type in Lauren classification and had invaded to the submucosa. Lymphatic metastasis was also confirmed (8/63). Thus, the patient had advanced gastric cancer with a dismal prognosis. Sample collection and sequencing analysis were approved by the ethics committee of Southern Medical University, and the patient provided written informed consent.

### Sample collection and DNA extraction

We harvested matched gastric cancer tissue and adjacent normal gastric mucosa, peritoneal metastasis and adjacent normal peritoneal tissue from one patient by laparoscopy. The DNeasy Blood & Tissue Kit (Qiagen, Germany) was used according to the manufacturer's instructions to extract and purify DNA from the harvested tissues. The concentration and quality of the DNA were determined using a NanoDrop ND-1000 spectrophotometer. Finally, 0.8%agarose gel electrophoresis was performed to confirm the quality of the DNA. Four DNA samples passed all of the strict quality supervision tests and were available for WES.

### Whole-exome capture

We selected IlluminaHiSeq 2500in a paired end 2×100nt multiplex procedure to capture all exons of the samples. First, we constructed a DNA library by fragmenting the genomic DNA. Second, we utilized a Qubit^®^ 2.0 Fluorometer to determine the concentration of the library, and the Agilent 2100 system was used to investigate the library's quality. Next, we utilized the Illumina PE Flow Cell v3 – HS system to sequence randomized DNA fragments. All sequencing processes were controlled by data collection software according to the HiSeq 2500 User Guide.

### Analysis of raw sequencing data

We aligned the paired-end reads to the reference human genome (hg19) using the third-party software BWA (Burrows–Wheeler Alignment, version5.9) with default parameters for the deletion of possible PCR repeats by samtools rmdup. The average mapping ratio was as high as 96%. The Flagstat tool was utilized to assess the mapping information. Next, we analyzed the distribution of each sample's reads in the target region and the enrichment of reads in the genome. The average sequencing depth in the exome region of the case was approximately 100×; under these conditions, at least 90% of the exome region was covered by 10 or more reads, and the coverage of the target region was approximately 80%(Supplementary Figure S1). SNVs (single nucleotide variations) and INDELs (insert and deletion mutations) were then processed using the GATK UnifiedGenotyper (GenomeAnalysisTK-3.1-1). Finally, we annotated the mutations using ANNOVAR software.

### Identification of somatic SNV and INDEL mutations

**Figure 6:**
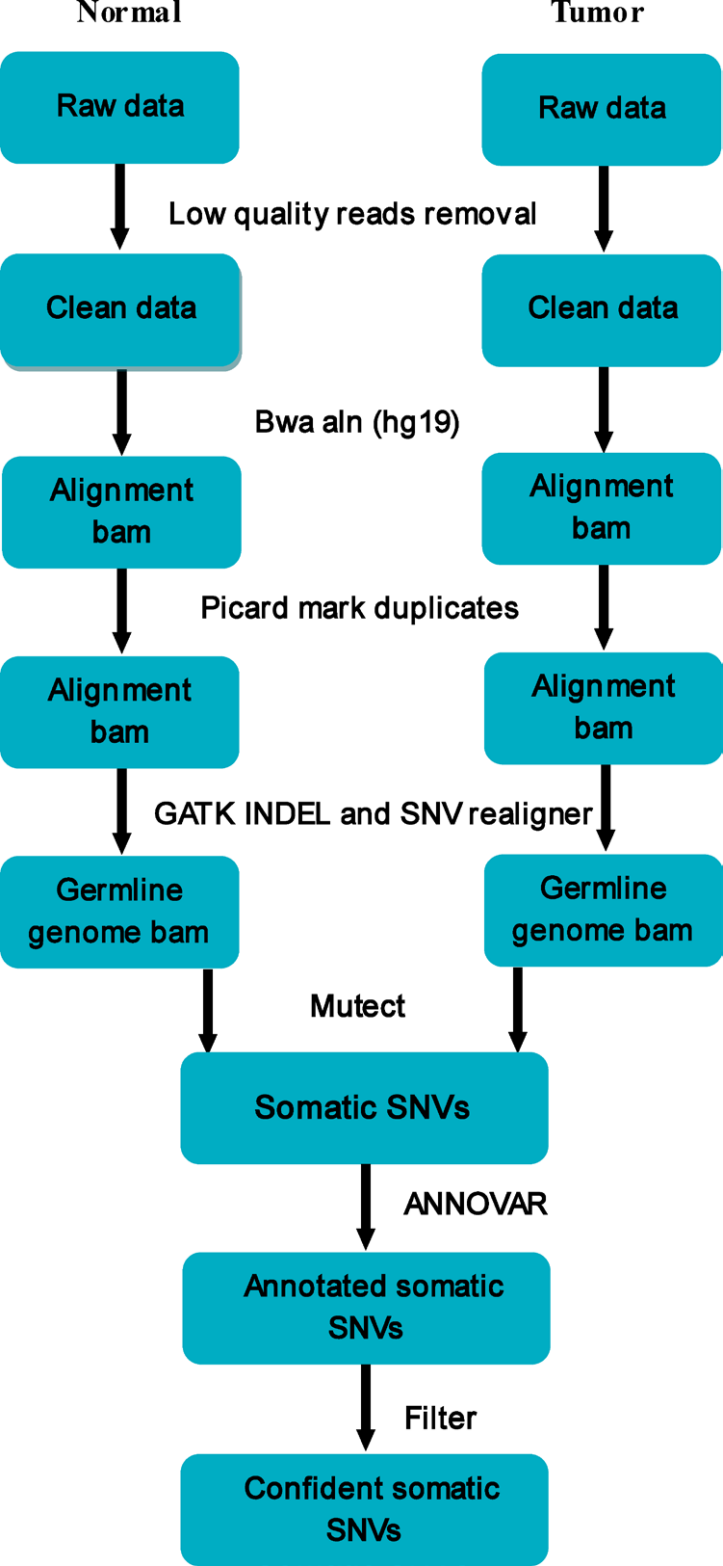
Pipeline of somatic mutation analysis from raw sequencing data Low-quality reads were removed, and bam was performed for alignment using GATK INDEL and SNV realigner after eliminating possible duplicates. MuTectwas used to identify somatic SNVs, and all somatic SNVs were annotated by ANNOVAR. By filtering false positives, confident somatic SNVs were obtained.

We applied MuTect to the WES data to detect somatic point and INDEL mutations. Normal gastric mucosa tissue and peritoneum tissue were used as references for somatic mutations of the primary and secondary tumors, respectively. Low-quality reads were first removed, bam was performed for alignment by GATK INDEL and SNV realigner after eliminating possible duplicates, and MuTect was then used to identify somatic SNVs. All somatic SNVs were annotated by ANNOVAR, which annotates by gene symbol, chromosome position, reference bases and observed bases, and mutation type. By filtering false positives, confident somatic SNVs were obtained (Figure 6).

### Confirmation of non-synonymous somatic mutations

We exploited the Verity 96-well PCRamplifier(ABI, USA) to perform PCR by adding special primers, followed by conventional PCR-based Sanger sequencing using the ABI3730XL(ABI, USA) sequencer, the gold standard of sequencing systems. Next, we compared the results with the next-generation sequencing data to confirm the non-synonymous somatic mutations.

### Comparison with a public database

COSMIC V76 is the latest version of the database of the catalog of somatic mutations in cancer. The database includes 1, 192,776 tumor samples for sequencing and25, 133whole-genome sequencing projects that have identified 3,942,175 coding mutations. Most of the data have been imported from the TCGA and ICGC databases. We compared our somatic mutations with the COSMIC database to identify driver genes of gastric cancer with peritoneal metastasis.

## SUPPLEMENTARY FIGURE


