# Supplementary material for: Whole-exome sequencing to identify somatic mutations in peritoneal metastatic gastric adenocarcinoma: A preliminary study
Source: Oncotarget. 2016 May 30;7(28):43894–906. doi: 10.18632/oncotarget.9707 (PMC5190066; doi:10.18632/oncotarget.9707)
Supplement: Supplementary file 1 [file oncotarget-07-43894-s001.pdf]

Whole-exome sequencing to identify somatic mutations in peritoneal metastatic gastric adenocarcinoma: A preliminary study

SUPPLEMENTARY FIGURE

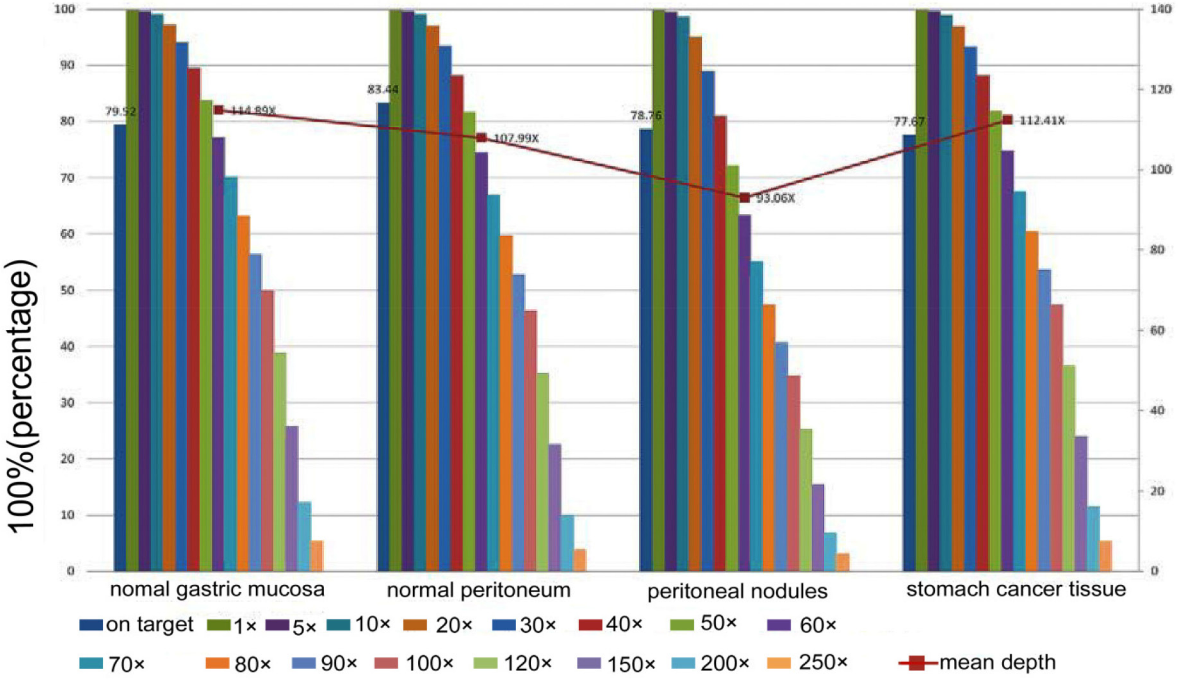

Supplementary Figure S1: The enrichment of on-target reads in genome. The first bar on the left in four group represent the percentage of on-target reads, indicates the efficiency of capture. The red folding line represents the mean coverage of our sequencing. The other bars indicate ratios of reads in different sequencing depth.
